# The Role of Heterotrimeric G-Protein Beta Subunits During Nodulation in *Medicago truncatula* Gaertn and *Pisum sativum* L.

**DOI:** 10.3389/fpls.2021.808573

**Published:** 2022-01-12

**Authors:** Andrey D. Bovin, Olga A. Pavlova, Aleksandra V. Dolgikh, Irina V. Leppyanen, Elena A. Dolgikh

**Affiliations:** ^1^All-Russia Research Institute for Agricultural Microbiology, Saint Petersburg, Russia; ^2^Department of Genetics and Biotechnology, Saint Petersburg State University, Saint Petersburg, Russia

**Keywords:** legume-rhizobial symbiosis, heterotrimeric G-protein, beta subunits, RNAi based suppression, promoter-GUS fusion localization, *Medicago truncatula* Gaertn, pea *Pisum sativum* L., co-immunoprecipitation

## Abstract

Heterotrimeric G-proteins regulate plant growth and development as master regulators of signaling pathways. In legumes with indeterminate nodules (e.g., *Medicago truncatula* and *Pisum sativum*), the role of heterotrimeric G-proteins in symbiosis development has not been investigated extensively. Here, the involvement of heterotrimeric G-proteins in *M. truncatula* and *P. sativum* nodulation was evaluated. A genome-based search for G-protein subunit-coding genes revealed that *M. truncatula* and *P. sativum* harbored only one gene each for encoding the canonical heterotrimeric G-protein beta subunits, MtG beta 1 and PsG beta 1, respectively. RNAi-based suppression of *MtGbeta1* and *PsGbeta1* significantly decreased the number of nodules formed, suggesting the involvement of G-protein beta subunits in symbiosis in both legumes. Analysis of composite *M. truncatula* plants carrying the *pMtGbeta1:GUS* construct showed β-glucuronidase (GUS) staining in developing nodule primordia and young nodules, consistent with data on the role of G-proteins in controlling organ development and cell proliferation. In mature nodules, GUS staining was the most intense in the meristem and invasion zone (II), while it was less prominent in the apical part of the nitrogen-fixing zone (III). Thus, MtG beta 1 may be involved in the maintenance of meristem development and regulation of the infection process during symbiosis. Protein–protein interaction studies using co-immunoprecipitation revealed the possible composition of G-protein complexes and interaction of G-protein subunits with phospholipase C (PLC), suggesting a cross-talk between G-protein- and PLC-mediated signaling pathways in these legumes. Our findings provide direct evidence regarding the role of MtG beta 1 and PsG beta 1 in symbiosis development regulation.

## Introduction

Heterotrimeric G-proteins are known to respond to a wide variety of external stimuli and interact with different cytosolic and membrane-associated effectors, therefore they may play an essential role in regulating plant growth and development as master regulators of signal transduction pathways (Urano and Jones, 2014; Pandey and Vijayakumar, 2018). Analysis of mutants showed that the heterotrimeric G-proteins in plants are involved in such processes as the control of organ development (Lease et al., 2001; Ullah et al., 2003; Ding et al., 2008; Mudgil et al., 2009), response to biotic and abiotic stress (Joo et al., 2005; Llorente et al., 2005; Delgado-Cerezo et al., 2012), hormonal regulation and signaling, and cell proliferation (Ullah, 2001).

Heterotrimeric G-proteins consist of G alpha, G beta, and G gamma subunits and are associated with the cell membrane in the inactive state. In animals and fungi, G alpha subunits are activated by several G-protein-coupled receptors (GPCRs); however, although single GPCRs were found in plants, their functional association with G-proteins was not shown (Urano and Jones, 2014; Roy Choudhury and Pandey, 2016). Recent studies have demonstrated that G-proteins in plants are activated by single-pass transmembrane receptor-like kinases (RLKs) or through the interaction between RLKs and seven-transmembrane domain regulators of G-protein signaling (RGSs) (Bommert et al., 2013; Choudhury and Pandey, 2013; Ishida et al., 2014; Tunc-Ozdemir et al., 2016). During signal perception, a conformational change stimulates the exchange of GTP for GDP on the G alpha subunit, promoting dissociation of the G beta-gamma complex. This event is required for signal transduction in plants and animals. In contrast to animals, the genomes of plants encode significantly less G-protein subunits. For example, *Arabidopsis* and rice contain only one G alpha, three extra-large G alpha (XLG), one G beta, and three G gamma subunits (Perfus-Barbeoch et al., 2004; Trusov and Botella, 2016).

Using a pharmacological approach, previous studies proposed the involvement of G-proteins in legume-rhizobial symbiosis regulation in legumes (Pingret, 1998); however, the exact mechanisms underlying this regulation and specific components of the G-protein network remain poorly characterized. Some effectors of G-protein signaling, such as phospholipase C (PLC) and D (PLD), are involved in mastoparan-induced root hair deformations during nodulation (Pingret, 1998; den Hartog et al., 2001, 2003; Charron et al., 2004; De Los Santos-Briones et al., 2009). Mastoparan is a well-known heterotrimeric G-protein agonist. Using a pharmacological approach, the relationships among G-protein activation, production of phospholipid metabolites by PLC and PLD, generation of calcium spiking, expression of specific *ENOD* genes, and root hair deformations were revealed in *Vicia sativa*, *Medicago sativa*, and *Medicago truncatula* (den Hartog et al., 2001, 2003; Charron et al., 2004; Sun et al., 2007).

In legumes with determinate types of nodules, such as soybean, a set of G alpha, G beta, and G gamma subunits were found using a genome-wide approach (Bisht et al., 2011) and some of them shown to be required for symbiosis development with rhizobia (Choudhury et al., 2011; Choudhury and Pandey, 2013, 2015). Moreover, the interaction between the G alpha subunits of soybean with lysin motif (LysM)-RLKs NFR1α and NFR1β (involved in Nod factor perception) was shown using the yeast split-ubiquitin system and bimolecular fluorescence complementation (Choudhury and Pandey, 2013). These findings were in line with previous hypotheses regarding the possible participation of G-proteins in signal transduction pathways activated upon perception of Nod factors.

In contrast, in legumes with indeterminate nodules, such as *M. truncatula* and *Pisum sativum*, in which nodule primordia develop in inner cortex and mature nodules retain a persistent meristem, the role of heterotrimeric G-proteins in symbiosis development has not been investigated in detail. However, several isoforms of G-protein subunits have been described in *P. sativum* and have been shown to be involved in the regulation of salinity and heat stress (Misra et al., 2007). Although heterotrimeric G-proteins are promising downstream components of the Nod factor perception pathway, their specific role in symbiosis development remains unclear. Advances in our understanding of G-protein networks will allow precise manipulation of legume plants to improve agronomically important traits. Here, RNAi-based suppression, promoter-β-glucuronidase (GUS) fusion, and co-immunoprecipitation were used to evaluate the involvement of the G-protein complex in the nodulation process in the model legume, *M. truncatula*, and the crop legume pea *P. sativum* (pea).

## Materials and Methods

### Bacterial Strains and Inoculation

Inoculation of *M. truncatula* plants was performed with the *Sinorhizobium meliloti* strain 2011. Pea plants *P. sativum* L. were inoculated with *Rhizobium leguminosarum* biovar *viciae* wild type strain CIAM1026. Bacterial liquid culture was grown in B^–^ medium (Van Brussel et al., 1977), diluted up to the optical density at 600 nm (OD_600_) 1.0 and applied to plants at the next day after planting.

### Plant Material and Growth Conditions

Seeds of *Medicago truncatula* cv Jemalong A17 were sterilized in concentrated sulfuric acid for 10 min followed by washing with sterile water six times at room temperature. After that the seeds were incubated in bleach for 2 min with subsequent rinsing with excess of sterile distilled water three times. Seeds were germinated on 0.8% water agar in Petri dishes and incubated at 4*^o^*C in 24 h for imbibition. After that the dishes were placed in inverted position in the dark at 23°C for germination and incubated for overnight.

Pea seeds of cultivar Finale were sterilized in concentrated sulfuric acid for 5 min followed by washing at least 4 times with excess of sterile distilled water. Seeds were germinated on sterile 1% water agar in Petri dishes for 5 – 7 days in the dark at 23°C.

### Constructs for RNAi Based Suppression of *G beta* Genes

Fragments of coding sequences of *MtGbeta1* or *PsGbeta1* genes (226 bp each, from 150 to 375 bp in both *MtGbeta1* and *PsGbeta1*) were amplified with specific primers flanked with *attB1* and *attB2r* sequences using Phusion Plus DNA Polymerase (Thermo Fisher Scientific, United States). Amplified fragments were separated by agarose gel electrophoresis and then purified from the gel using Cleanup standard kit (Evrogen, Russia) according to the user manual. Purified fragments were cloned into gateway pDONR221 vector using BP clonase (Thermo Fisher Scientific, United States). Chemically competent *E. coli* TOP10 cells were used for transformation, selection was performed on LB medium containing kanamycin 100 μg/ml. After checking by sequencing, the pDONR221 vectors with cloned fragments were used for recombination in pK7GWIWG2II destination vector (Ghent University, Belgium) using LR clonase (Thermo Fisher Scientific, United States). Transformants were grown on selective LB medium containing spectinomycin 75 μg/ml and chloramphenicol 25 μg/ml. Final constructs were checked by sequencing.

### Protein Synthesis in *Escherichia coli* and Co-immunoprecipitation Assay

Full-length coding sequences of *MtGbeta1, PsGbeta1, MtGalpha1, MtGalpha2, PsGalpha1, PsGalpha2* as well as *MtPLC1, PsPLC1*, *MtPLD1, PsPLD1* genes were amplified with specific primers flanked with the sequences of restriction sites (Supplementary Table 1) using Phusion Plus DNA Polymerase (Thermo Fisher Scientific, United States). After purification from the gel, the full-length coding sequences were cloned in the frames into pRSETa-6xHIS or/and pRSETa-3xFLAG vectors using T4 ligase. Transformants were grown on selective LB medium containing ampicillin 100 μg/ml. Constructs were checked by sequencing. Electrocompetent *E. coli* C41 were transformed with constructs in pRSETa-6xHIS or pRSETa-3xFLAG vectors using Gene Pulser XCell electroporation system (Bio-Rad Laboratories, United States). Transformants were grown on selective solid LB medium containing ampicillin 100 μg/ml for overnight, then a few fresh colonies were transferred into a big flask containing 200 ml LB with ampicillin 100 μg/ml. Suspension was grown up to OD_600_ = 0.6 at 37°C in shaker and then synthesis was induced by addition 0.5 mM IPTG. After 2 h of cultivation at 28°C with intensive shaking, the cells were pelleted by centrifugation at 3000 g for 15 min in 50 ml Falcon tubes. Supernatant was removed thoroughly and cell pellets in tubes were stored at −80°C.

Frozen bacterial cells containing synthesized proteins were thawed and resuspended in lysis buffer (2 ml per pellet from 50 ml of culture) with protease inhibitor cocktail INHIB1-1KT (Merck, Germany) (0.1 mM AEBSF and 1 μg/ml of each leupeptin, aprotinin, antipain, pepstatin and chymostatin). Samples were aliquoted in two 1.5 ml tubes (1 ml) and sonicated 3 times for 30 s with 30 s pauses on ice. After sonication the lysates were centrifugated at 20000 g for 25 min. Soluble protein fractions were collected and used for co-immunoprecipitation. Co-immunoprecipitation was carried out using a μMACS kit (Miltenyi Biotec, Germany) containing MicroBeads with immobilized anti-HIS or anti-FLAG antibodies. Pairs of proteins with appropriate tags were placed in tubes with 50 μl of μMACS MicroBeads and incubates with slow shaking at 4°C for 1.5 h. Protein mixture and lysis buffer (used for column washing) were degassed by vacuum for 5 min on ice and then applied to μMACS columns according to the user manual. Washing was performed using lysis buffer in total volume of 1 ml. Elution performed according to the user manual. Eluted proteins were separated in 12% polyacrylamide gels and then transferred to nitrocellulose membrane for subsequent Western blot analysis.

### Promoter Fusion Analysis

2700 bp fragment upstream of start codon of the *MtGbeta1* gene was amplified with specific primers containing *attB1* and *attB2r* sequences using two-step protocol (98°C 0:30, 60°C 2:30, 30 cycles) with Phusion Plus DNA Polymerase (Thermo Fisher Scientific, United States). After purification from the gel, the promoter was cloned into pDONR221 vector by BP clonase (Thermo Fisher Scientific, United States). Finally, the promoter was subcloned into pBGWFS7.0 destination vector using LR clonase (Thermo Fisher Scientific, United States). *E. coli* TOP10 cells were used for chemical transformation and selection was done on LB medium containing spectinomycin 50 μg/ml.

### *Agrobacterium rhizogenes* Mediated Plant Transformation

5 – 7 days-old pea seedlings of cv. Finale were transferred to light into sterile dark plastic boxes with liquid Jensen’s medium (1 g/l CaHPO_4_, 0.2 g/l K_2_HPO_4_, 0.2 g/l MgSO_4_, 0.2 g/l NaCl, 7.34 mg/l Na-Fe-EDTA, 87.5 mg/l CuSO_4_ × 5H_2_O, 1.16 mg/l MnSO_4_ × 7H_2_O, 2.4 mg/l ZnSO_4_ × 7H_2_O, 3.17 mg/l H_3_BO_3_, 1 mg/l Na_2_MoO_4_ × 2H_2_O), which were placed in a big plastic vessel (Sac O2, Belgium). Seedlings were cultivated in the growth chamber for 5 – 7 days (+ 21°C, humidity 60%, 16 h/8 h light/darkness) until the stage of two internodes. Seedlings were cut at the hypocotyl region and transformed with freshly grown *Agrobacterium rhizogenes* strain ARqua 1 carrying an appropriate construct. After transformation plants were placed on solid Jensen’s medium (3 – 4 plants per box) in round plastic boxes with green filter (E1674, Duchefa, The Netherlands). Lower parts of plants were covered with wet cotton wool and aluminum foil and cultivated for 10 – 14 days until callus is appeared (Leppyanen et al., 2019). After that seedlings were transferred to Emergence medium (3 mM MES pH 5.8, 2.5 g/l KNO_3_, 0.4 g/l, MgSO_4_ × 7H_2_O, 0.3 g/l NH_4_H_2_PO_4_, 0.2 g/l CaCl_2_ × 2H_2_O, 10 mg/l MnSO_4_ × 4H_2_O, 5 mg/l, H_3_BO_3_, 1 mg/l ZnSO_4_ × 7H_2_O, 1 mg/l KI, 0.2 mg/l CuSO_4_ × 5H_2_O, 0.1 mg/l, NaMoO_4_ × 2H_2_O, 0.1 mg/l CoCl_2_ × 6H_2_O, 15 mg/l FeSO_4_ × 7H_2_O, 20 mg/l Na_2_EDTA, 100 mg/l myoinositol, 5 mg/l nicotinic acid, 10 mg/l pyridoxine HCl, 10 mg/l thiamine HCl, 2 mg/l glycine, 1% sucrose, 1% Gelrite agar) containing 150 μg/ml cefotaxime and incubated for additional 3 – 4 days. Transgenic roots were selected by visualization of DsRED or GFP (green fluorescent protein) expression. Plants were transferred into pots with vermiculite saturated with Jensen’s medium containing 1.5 mM NH_4_NO_3_.

1 days – old *M. truncatula* cv. Jemalong A17 seedlings were transferred on Fahreus agar plates [60 mg/l MgSO_4_ × 7H_2_O, 50 mg/l KH_2_PO_4_, 78 mg/l Na_2_HPO_4_, 7.34 mg/l Na-Fe-EDTA, 83 mg/l Ca(NO_3_)_2_, 66 mg/l CaCl_2_, 87.5 mg/l CuSO_4_ × 5H_2_O, 1.16 mg/l MnSO_4_ × 7H_2_O, 2.4 mg/l ZnSO_4_ × 7H_2_O, 3.17 mg/l H_3_BO_3_, 1 mg/l Na_2_MoO_4_ × 2H_2_O], roots were covered with wet filter paper and incubated in the growth chamber (+ 21°C, humidity 60%, 16 h/8 h day/night) for 48 h. Plants were cut off at the hypocotyl region and transformed with *A. rhizogenes* Arqua1 strain carrying a necessary construct. Plants were incubated on Fahreus medium with roots positioned between two wet filter papers for 7 days until calli are appeared. After that they were transferred to plates with Emergence medium containing 150 μg/ml cefotaxime and incubated for additional 7 days or more. Plants with transgenic roots were transferred to vermiculite saturated with Farheus medium.

The fragments of roots without nodules (about 100 mg) or nodules (10 – 15 nodules per probe) were harvested from the plants and used for subsequent gene expression analysis. In experiments on RNAi based suppression and promoter fusion analysis two or three biological replicates were analyzed with 15 – 20 plants in each group.

### Quantitative Reverse Transcription–Polymerase Chain Reaction (qRT-PCR) Analysis

Total RNA was isolated from frozen roots. Material was ground with a mortar and pestle to a fine powder in liquid nitrogen and extracted with Trizol reagent (Bio-Rad Laboratories, United States). 1 μg of total RNA was used to synthesize cDNA with the RevertAid Reverse Transcriptase (Thermo Fisher Scientific, United States). cDNA samples were diluted to a total volume of 100 μl. Quantitative real-time PCR was performed using Bio-Rad iQ Sybr master mix (Bio-Rad Laboratories, United States) following the manufacturer’s recommendations and run on a CFX-96 real-time PCR detection system with C1000 thermal cycler (Bio-Rad Laboratories, United States). The threshold cycle (Ct) values were calculated using the Bio-Rad CFX Manager 1.6 program and analyzed using the 2-ΔΔCt method. List of primers was presented in Supplementary Table 1.

### Glucuronidase Staining of Material

Roots with nodules were thoroughly washed in tap water. Parts of roots with primordia or nodules were degassed under a vacuum (-0.8 bar; ME 1C vacuum pump, Vacuubrand) for 5 min in 100 mM sodium phosphate buffer (pH 7.0). For GUS staining the samples were incubated in 100 mM sodium phosphate buffer (pH 7.0), containing 1% Triton-X-100, 1 mM X-Gluc, 1 mM EDTA (pH 8.0), 0.5 mM potassium ferricyanide, 0.5 mM potassium ferrocyanide for 4 – 24 h until staining development. For fixation plant material was placed in phosphate saline buffer (5 mM KH_2_PO_4_, 140 mM NaCl, 2.7 mM KCl, Na_2_HPO_4_ 6.5 mM).

### Phylogenetic Reconstruction

For identification of homologous of previously detected *Galpha*, *Gbeta* and *Ggamma A. thaliana* and *G. max* genes in *P. sativum* and *M. truncatula* genomes BLASTP analysis (v 2.6.0, word_size = 3, evalue < 10e-20) was performed. Sequences selected during such analysis were investigated for the presence of specific G-protein conserved domains (Urano et al., 2013) using InterProScan web server (Jones et al., 2014).

First of all, for phylogenetic reconstruction amino acid sequences of selected genes were aligned using MAFFT tool v7.453 (Katoh and Standley, 2013). Based on these alignments, a phylogenetic tree was constructed using the Maximum-Likelihood method with help of the IQ-TREE web server (Trifinopoulos et al., 2016). The bootstrap values were obtained from 1000 bootstrap replicates of Ultrafast Bootstrap (Minh et al., 2013). For visualization of resulted trees R package ggtree was used (Yu, 2020).

### Assessment of *Galpha, Gbeta* and *Ggamma* Gene Expression in *Medicago truncatula*

Raw reads from RNA-Seq project PRJNA552042 (Schiessl et al., 2019) including data for 24, 48, 72, 96,120, and 168 h after inoculation or mock treatment for WT plants were used for analysis. The *M. truncatula* genome version 4.0 was used as a reference. The reads were mapped to the genome using HISAT2 (Kim et al., 2019) tool v 2.1.0 with default parameters, and raw counts were obtained by FeatureCounts from Subreasd package (Liao et al., 2014). The edgeR package was used to calculate CPM values. Expression data for nodules was obtained from Pecrix et al. (2018). For data visualization ggplot2 (Wickham, 2016) package was used and Adobe Illustrator was implemented for final figures assemblies.

### Statistical Methods and Computer Software

One-way ANOVA and Tukey’s test were used to compare gene expression levels.

## Results

### *Medicago truncatula* and *Pisum sativum* Genomes Encode a Set of G-Protein Alpha, Beta, and Gamma Subunits

A genome-based search for genes encoding G-protein subunits in the model legume *M. truncatula* and the crop legume *P. sativum* was performed. Briefly, amino acid sequences of *Arabidopsis thaliana* and *Glycine max* orthologs were used for BLASTP analysis (Camacho et al., 2009), after which, specific domains were identified using InterProScan (Jones et al., 2014).

**FIGURE 1:**
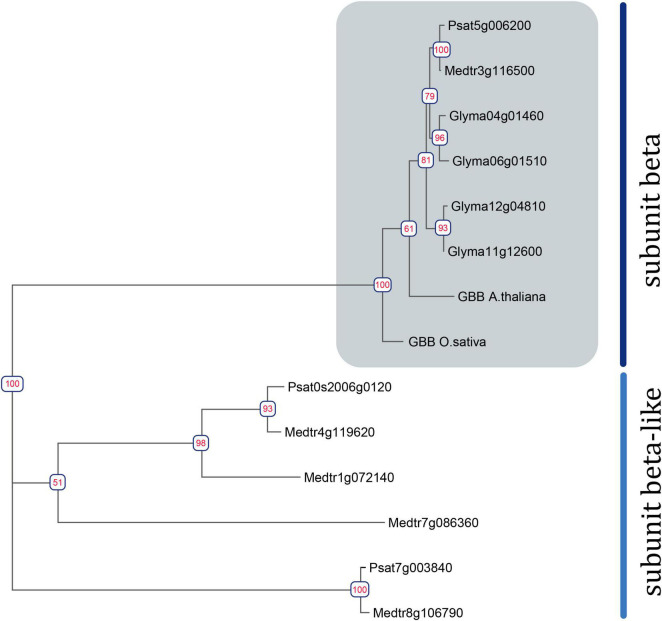
Phylogenetic tree constructed using the Maximum-Likelihood method based on amino acid sequences of *G. max* and *A. thaliana Gbeta* genes and their homologous identified in *P. sativum* and *M. truncatula* genomes. Numeric values indicate branch support based on 1000 UltraFast bootstrap replicates.

The search revealed two *Galpha*, three *extra-large Galpha (XLG)*, one *Gbeta*, and five *Ggamma* genes in the *M. truncatula* genome (sequence v4) (Tang et al., 2014), and two *Galpha*, three *XLGs*, one *Gbeta*, and six *G gamma* genes in the *P. sativum* genome (sequence v1) (Figure 1 and Supplementary Figures 1, 2) (Pecrix et al., 2018). Structurally, canonical G beta subunits contain an N-terminal Coil domain and at least seven WD40 domains, which form a beta-propeller structure (Urano et al., 2013). Only one gene encoding a G beta subunit with the latter structure was found in the genomes of *M. truncatula* and *P. sativum* genomes. However, at least four *MtGbeta*-like and two *PsGbeta*-like genes exist in the genomes of these legumes and may be of interest for future research. These proteins lack an N-terminal coil domain as well as family specific domains; however, they do contain at least seven WD40 domains.

To assess the homology between the identified G beta subunits and those of well-studied model plants (*A. thaliana*, *Oryza sativa, G. max*), a phylogenetic tree was constructed (Figure 1). Phylogenetic trees were also constructed for *G alpha, G gamma*, and *XLG* genes (Supplementary Figures 1, 2).

To evaluate the expression levels of *G alpha*, *G beta*, and *G gamma* in *M. truncatula* roots after inoculation, a publicly available RNAseq dataset was analyzed (GSE133612) (Schiessl et al., 2019). This particular dataset encompasses expression data across early stages of symbiosis development. In addition, an RNAseq dataset for nodules was also analyzed (Roux et al., 2014; Pecrix et al., 2018). *In silico* analyses revealed that *MtGbeta1* (Medtr3g116500) expression was relatively high in inoculated roots and nodules and non-inoculated roots. Moreover, a significant upregulation in the expression of *MtGalpha1* (Medtr1g015750) was detected in inoculated roots at the early stages of symbiosis development and in nodules, when compared to control roots (Supplementary Figure 3). However, *MtGalpha2* (Medtr3g105240) was not expressed in roots or nodules (Supplementary Figure 3).

### The Heterotrimeric G-Protein Beta 1 Subunit Positively Regulates Nodulation in *Medicago truncatula* and *Pisum sativum*

**FIGURE 2:**
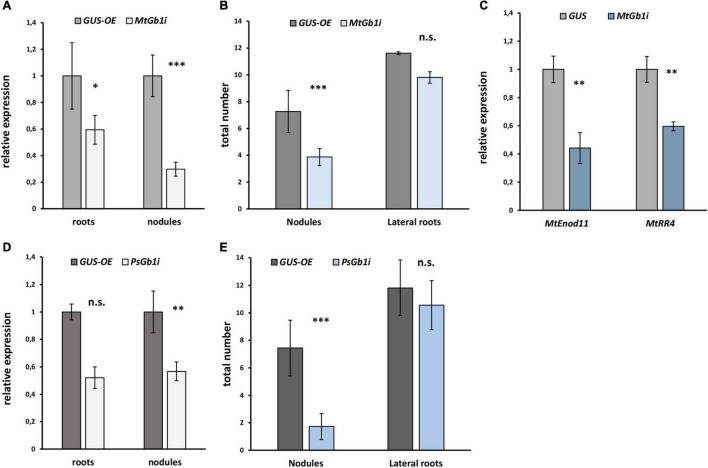
The effect of *Gbeta1* gene suppression on root development and nodulation in *M. truncatula* A17 **(A,B)** and *P. sativum* cv. Finale **(D,E)** plants. Composite plants with the *Gbeta1* gene suppression in transgenic roots (*Gbeta*-RNAi) were compared with control plants with β-glucuronidase gene overexpression (*GUS*-OE). Analysis was performed two weeks after inoculation (2 wai) (*M. truncatula*) and 3 weeks after inoculation (3 wai) (*P. sativum*). Approximately 50% suppression of the *Gbeta1* gene in the transgenic roots and nodules of both legumes was revealed **(A,D)**. The number of nodules in transgenic fluorescent roots of *Gbeta*-RNAi plants was significantly reduced **(B,E)**, whereas the total number of transgenic fluorescent lateral roots did not change in both cases. Expression of early symbiotic markers, the *MtEnod11* and *MtRR4*, in the transgenic hairy roots of *MtGbeta1*-RNAi plants **(C)**. Two biological replicates were analyzed for *M. truncatula* and *P. sativum*, each contained 15 – 20 plants per variant. In case of *M. truncatula* totally 47 and 26 transgenic fluorescent roots of *MtGbeta*-RNAi and *GUS*-OE plants, correspondently, were included in the analysis, while 29 and 16 transgenic fluorescent roots of *PsGbeta*-RNAi and *GUS*-OE plants were used in *P. sativum*. The number of nodules were scored only in transgenic fluorescent roots. Values are means ± SEM. *, Significant difference at *P* ≤ 0.05; ^**^, Significant difference at *P* ≤ 0.01; ^***^, Significant difference at *P* ≤ 0.01; ns, non-significant difference.

Since the complex of G beta/gamma subunits may play an important role in signal transduction following signal perception, the remainder of the study focused on the *G beta* genes. Since only one typical G beta 1 subunit was found in both *M. truncatula* (*MtGbeta1*) and *P. sativum* (*PsGbeta1*), the role thereof was evaluated on symbiosis. To silence the *Gbeta1* genes in *M. truncatula* and *P. sativum*, an RNAi approach was employed. Composite plants were obtained with approximately 50% suppression of the *Gbeta1* gene in the transgenic roots and nodules of both legumes (Figure 2). As controls, plants expressing beta-galactosidase under the p35S promoter (GUS-OE) were used. Comparative analyses showed a statistically significant decrease in nodule number in the transgenic fluorescent roots in RNAi lines of both legumes, while other parameters such as number of lateral roots did not change (Figures 2A-E and Supplementary Figure 4). It correlated with decreasing the expression of early symbiotic markers such as *MtEnod11* and *MtRR4* in the transgenic hairy roots of *Gbeta1* -RNAi plants (Figure 2C). The results suggested that *MtGbeta1* in *M. truncatula* (Figures 2A,B and Supplementary Figure 4) and *PsGbeta1* in *P. sativum* (Figures 2D,E) were required for symbiosis development in legumes with indeterminate type of nodules.

### The *MtGbeta1* Promoter Is Active at the Various Stages of Symbiosis Development

**FIGURE 3:**
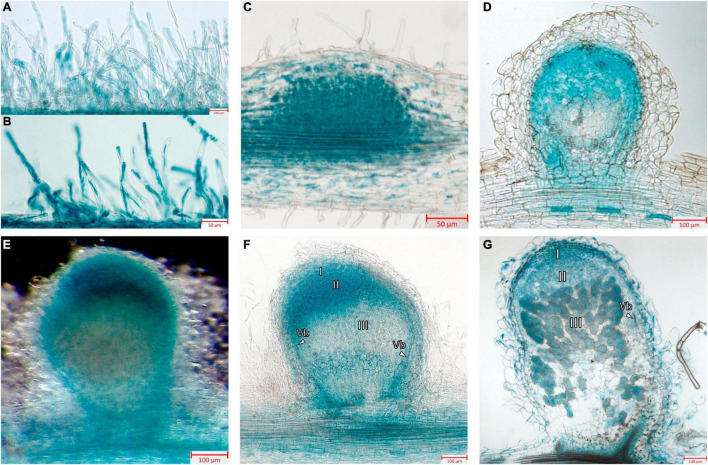
Localization of *MtGbeta1* expression in *M. truncatula* non-inoculated and inoculated roots, nodule primordia and 2-week-old nodules carrying a *pMtGbeta1:GUS* genetic construct. **(A)** Non-inoculated roots. **(B)** Inoculated roots. **(C)** Nodule primordia (50 μm section). **(D)** Young nodule (50 μm section). **(E)** A general view of the nodule. Image was done using dark field like illumination **(E–G)** Two-week-old nodules (50 μm section). I, meristem zone; II, infection zone; III, nitrogen fixation zone; arrows indicate vascular bundles (Vb). Scale bars are 50 μm **(B,C)** and 100 μm **(A,D–G)**.

Next, the promoter activity of the *MtGbeta1* gene was analyzed in the roots of composite plants expressing *pMtGbeta:GUS* at different stages following *Sinorhizobium meliloti* 2011. At the early stages of symbiosis development, GUS staining was detected in the root hairs; however, the intensity of the signal was comparable to that of non-inoculated plants (Figures 3A,B). In the developing primordia of nodules and in young nodules appearing above the surface of roots, strong GUS staining was observed (Figures 3C,D). Staining was also observed in the primordia of lateral roots (data not shown). In mature nodules [2 weeks after inoculation (wai)] (Figures 3E–G), GUS staining was most intense in the meristem and invasion zone (II), while the signal was significantly weaker in interzone II-III, and in the apical part of the nitrogen-fixing zone (III) (Figures 3E,F). Although a signal was observed in the central part of nodules, it had a variable pattern, having a less or more pronounced intensity in the nitrogen-fixing zone (III) depending on the length of staining time and the level of transgene expression (Figure 3G). Finally, *pMtGbeta:GUS* expression was also observed in the vascular bundles.

### G Beta 1 and G Alpha Subunits Interact in Both *Medicago truncatula* and *Pisum sativum*

Although the G beta subunits of the complex are predominantly connected to signal transduction in the cell, the activation of the complex is achieved by G alpha subunits due to their association with GDP or GTP and their release from the complex. A BLASTP search revealed two G alpha subunits in *M. truncatula*, MtG alpha 1 (Medtr1g015750) and MtG alpha 2 (Medtr3g105240), which showed a high level of homology to two PsG alpha subunits, PsG alpha 1 (AF537218) and PsG alpha 2 (AF533438) previously identified in *P. sativum* (Misra et al., 2007). However, expression analysis showed that *MtGalpha2* was not expressed in roots and nodules (Supplementary Figure 3), and therefore, MtG alpha 2 was omitted from co-immunoprecipitation experiments.

**FIGURE 4:**
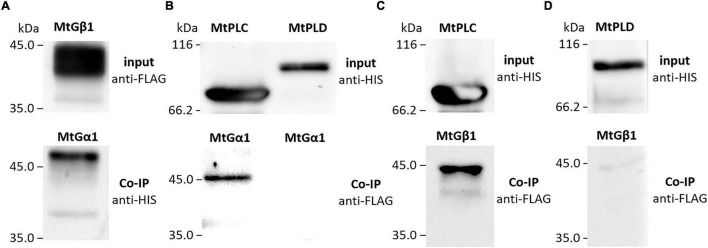
The result of co-immunoprecipitation of heterotrimeric G-protein subunits and phospholipases C (MtPLC) and D (MtPLD) from *M. truncatula*. A heterologous protein expression was carried out in *Escherichia coli* C41, allowing high levels of expression of all subunits of interest (MtG beta1, MtG alpha 1, MtPLC, MtPLD) fused to either a 6xHIS or a 3xFLAG tag. The assays revealed interactions between MtG beta 1 and MtG alpha 1 **(A)** as well as MtG alpha 1 and MtPLC **(B)**, MtG beta 1 and MtPLC **(C)**. No interactions were detected between any of the G-protein subunits and MtPLD **(B,D)**.

**FIGURE 5:**
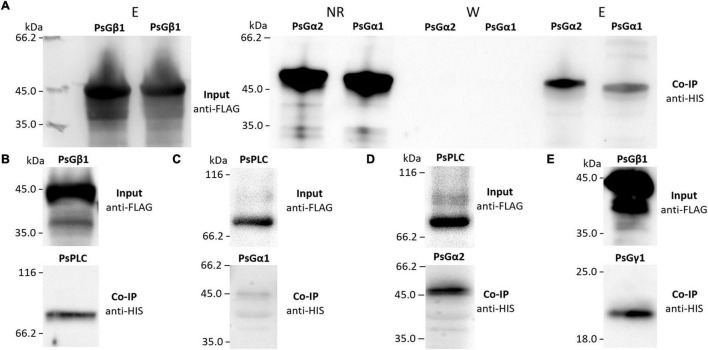
The result of co-immunoprecipitation of heterotrimeric G-protein subunits and phospholipases C (PsPLC) and D (PsPLD) from *P. sativum*. A heterologous protein expression was carried out in *E. coli* C41. Co-immunoprecipitation of PsG beta 1 with PsG alpha 1 and PsG alpha 2 **(A)** as well as PsG beta 1 with PsPLC **(B)**. PsPLC interacts with PsG alpha 2, but does not interact with PsG alpha 1 **(C,D)**. The interaction was detected between PsG beta 1 and PsG gamma 1 **(E)**. Eluted proteins were separated in 12% polyacrylamide gels and then transferred to nitrocellulose membrane for subsequent Western blot analysis. E – elution, NR – non-retained fraction, W – washing.

To verify the interactions between the G beta 1 and G alpha subunits, *in vitro* co-immunoprecipitations were performed. A heterologous protein expression was carried out in *Escherichia coli* C41, allowing high levels of expression of all subunits of interest (MtG beta1, PsG beta 1, MtG alpha 1, PsG alpha 1, and PsG alpha 2) fused to either a 6xHIS or a 3xFLAG tag (Figures 4, 5). Subunits were co-incubated and complexes purified using a μMACS column with antibodies. When MtG beta 1 and MtG alpha1 were co-incubated, both proteins were identified in the eluate. Moreover, PsG beta 1 co-eluted with both PsG alpha 1 and PsG alpha 2, as revealed by western blot analysis (Figure 5). However, the interaction was much stronger between PsG beta 1 and PsG alpha 2. Therefore, both pea PsG alpha 1 and PsG alpha 2 subunits may co-precipitate with PsG beta 1 protein and can be potential participants in heterotrimeric G-protein complex, while in *M. truncatula* the formation of one complex between MtG beta 1 and MtG alpha1 was shown.

### Phospholipase C Interacts With G Alpha and G Beta Subunits in Both *Medicago truncatula* and *Pisum sativum*

The involvement of PLC and PLD in the regulation of nodulation was previously described using a pharmacological approach (Charron et al., 2004). In the same study, PLC and PLD were suggested to be connected with heterotrimeric G-protein activation. The genome of *M. truncatula* encodes nine *PLC* and fifteen *PLD* genes (Supplementary Table 2), but we searched for significantly activated genes at the early stages of symbiosis.

Analysis of transcriptomic datasets for *M. truncatula* roots inoculated with rhizobia, or treated with Nod factors (van Zeijl et al., 2015; Damiani et al., 2016; Schiessl et al., 2019), revealed that the expression of *MtPLC1* (Medtr3g070560, MtrunA17_Chr3g0113471) (Damiani et al., 2016; Schiessl et al., 2019) and *MtPLD1* (Medtr4g010650, MtrunA17_Chr4g0003411) increased at the early stages of symbiosis development (Supplementary Figures 5, 6 and Supplementary Table 2). Genes in *P. sativum* were selected based on homology to the orthologs of *M. truncatula* and included one *PsPLC1* (Psat5g128400) gene and one *PsPLD1* (Psat7g255400) gene (Supplementary Table 2).

The respective coding sequences of *MtPLC, MtPLD* and *PsPLC, PsPLD* were cloned into pRSETa-6xHIS and pRSETa-3xFLAG, and proteins were expressed as before for co-immunoprecipitation assays. The assays revealed interactions between MtG beta 1 and MtPLC, and MtG alpha 1 and MtPLC (Figures 4, 5). Moreover, PsG beta 1 was able to interact with PsPLC, while only PsG alpha 2 could interact with PsPLC (Figures 4, 5). No interactions were detected between any of the G-protein subunits and MtPLD. It seems like either MtPLD do not interact with components of G-protein complex or more complicated regulation through additional regulators may be take place. At the same time, we could not exclude an incorrect folding of such big proteins (91.5 kDa) as MtPLD in heterologous system. Finally, PsPLD expression in the bacterial system failed; hence, its interaction with G-protein subunits could not be examined.

### Kinase Domain of LysM-Containing Receptor-Like Kinase K1 May Interact With G alpha Subunit *in vitro*

**FIGURE 6:**
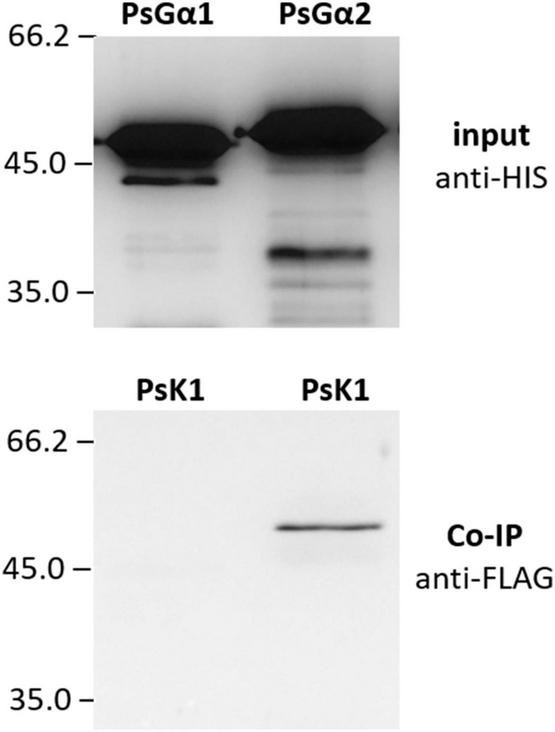
The result of co-immunoprecipitation between LysM-RLK PsK1 kinase domain and PsG alpha 1 and PsG alpha 2 subunits. LysM-RLK PsK1 interacts with PsG alpha 2, but does not interact with PsG alpha 1.

G-protein activation is mainly maintained by single-pass transmembrane RLKs. In *P. sativum*, at least two RLK complexes may be involved in the perception of Nod factors, which are activated at different stages of symbiosis development, PsSYM10/PsK1 and PsSYM10/PsSYM37 (Zhukov et al., 2008; Kirienko et al., 2018). In the complex PsSYM10/PsK1, the RLK PsK1 possesses an active kinase domain and seems to be required for signal transduction during early stages of pea-rhizobial symbiosis development (Zhukov et al., 2008; Kirienko et al., 2018). Here it was demonstrated using a co-immunoprecipitation assay that the kinase domain of PsK1 RLK interacts with PsG alpha 2, but not PsG alpha 1 (Figure 6). It may suggest the involvement of G-protein in the control of early stages of symbiosis development.

## Discussion

The main goal of this study was to analyze the role of the heterotrimeric G-protein beta subunits as the most important subunits for signal transduction in the cell during symbiosis development in *M. truncatula* and *P. sativum*. A genome-based search for genes encoding G-protein subunits revealed that the *M. truncatula* and *P. sativum* genomes encode one gene each for the canonical G beta subunit, MtG beta 1 and PsG beta 1, respectively. These subunits contain seven WD40 motifs and a coiled-coil motif at their N-terminal ends. These findings are in line with previous data for *P. sativum* (Misra et al., 2007). Moreover, additional *Gbeta*-like genes were found in the genomes of *M. truncatula* and *P. sativum*.

RNAi-based suppression of the *MtGbeta1* and *PsGbeta1* resulted in a significant decrease in the number of nodules formed. This suggests that these G beta 1 subunits of heterotrimeric G-proteins have a positive effect on the development of symbiosis in both legume plants, which belong to the same group. Moreover, the results of our experiments based on RNA interference were in line with those obtained in *G. max* for *GmGbeta1-4* genes, all having a high level of homology with *MtGbeta1/PsGbeta1* (Choudhury and Pandey, 2013). These results indicate that *MtGbeta1* and *PsGbeta1* are required for symbiosis development in legumes with indeterminate types of nodules. Further research, however, is necessary regarding their influence at specific stages of this symbiosis development.

Composite plants expressing *GUS* under the *MtGbeta1* promoter allowed insight into the activity of the *MtGbeta1* promoter. During the early stages of symbiosis development, intensive GUS staining was observed in the developing nodule primordia and young nodules. Staining was also observed in the primordia of lateral roots. *Gbeta1* promoter activity fits well with the data on the participation of G-proteins in the control of organ development and cell proliferation. As shown previously in *Arabidopsis*, G-proteins may influence cell cycle regulation through modulation of auxin transport (Lease et al., 2001; Chen et al., 2006; Mudgil et al., 2009). In mature nodules, the localization of GUS staining was the most intense in the meristem and invasion zone (II), while the signal was less prominent in interzones II-III and in the apical part of the nitrogen-fixing zone (III). These results suggest that MtG beta 1 may be involved in the maintenance of meristem development and regulation of the infection process. The exact mechanisms behind this still needs further research. The localization in vascular bundles may be related to regulation of the nutrients translocating via the vasculature.

To elucidate the composition of the heterotrimeric G-protein complex in *M. truncatula* and *P. sativum*, the corresponding G-proteins were expressed in *E. coli*, and their interactions analyzed using *in vitro* co-immunoprecipitations. The interaction between MtG beta 1 and MtG alpha 1, as well as MtG beta 1 and MtG gamma 1, was shown in our experiments. These results suggest the existence of a heterotrimeric G-protein complex comprising the MtG alpha 1/MtG beta 1 subunits in *M. truncatula*. Concurrently, two putative complexes consisting of PsG beta 1 and PsG alpha 1, and PsG beta 1 and PsG alpha 2 may exist in *P. sativum*. Hence, both *P. sativum* PsG alpha 1 and PsG alpha 2 subunits interact with PsG beta 1, serving as potential participants in the heterotrimeric G-protein complex, while in *M. truncatula*, the formation of a single complex between MtG beta 1 and MtG alpha1 takes place.

Signal transduction pathway activation depends on Nod factor perception by a few receptor complexes in *M. truncatula* and *P. sativum* (Ardourel et al., 1994; Geurts et al., 2005; Kirienko et al., 2018). Since G beta subunits usually do not bind to receptors themselves, the interaction between receptors to Nod factors and G alpha subunits were analyzed. In *P. sativum*, the interaction between LysM-RLK K1 – the Nod factor receptor, which controls the earliest reactions after signal perception (Kirienko et al., 2018) – and the PsG alpha 2 subunit was demonstrated. In accordance with these results, it was previously shown that the LysM-RLKs in soybean, GmNFR1a and GmNFRb, were able to interact with GmG alpha subunits. This suggests that heterotrimeric G-proteins may play an important role in plant response to Nod factor perception in legume plants during the early stages of symbiosis development.

Subsequent signal transduction in legume-rhizobial symbiosis may be connected to the activation of PLC and PLD by heterotrimeric G-protein subunits, as shown by previous studies using inhibitors (Pingret, 1998; den Hartog et al., 2003). Analysis of *M. truncatula* transcriptomic datasets of roots inoculated with rhizobia or treated with Nod factors (van Zeijl et al., 2015; Damiani et al., 2016; Schiessl et al., 2019) allowed the identification of *PLC* and *PLD* genes induced at the early stages of symbiosis. *In vitro* co-immunoprecipitation assays showed that MtG beta 1 and MtG alpha 1 interacted with MtPLC. Similarly, PsG beta 1 interacted with homologous PsPLC, while among the two alpha subunits, only PsG alpha 2 formed a complex with PsPLC and not PsG alpha 1. These results demonstrate possible cross-talk between G-protein- and PLC-mediated signaling pathways in legumes with indeterminate nodule types. It is also important to note that during heat and salinity stress in *P. sativum*, G-protein subunits interact with other forms of PsPLC (Y15253) (Misra et al., 2007), suggesting the specificity of signal transduction pathways in the activation in different processes.

Although the involvement of PLD in the regulation of root hair deformations was shown in *M. truncatula*, no interactions between PLD and G subunits were detected in both legumes. However, it could not be excluded that the interaction may be indirect through the stimulation of PLD by intracellular signals such as calcium.

In legume plants, two types of calcium reactions take place in response to the perception of Nod factors. These include calcium influx into the cells and calcium spiking in the nucleus and perinuclear space at the early stages of symbiosis (Shaw and Long, 2003). It was previously shown that different signaling pathways may be involved in the activation of calcium influx and calcium spiking. Perception of Nod factors activates the signaling pathway, including a receptor kinase with leucine reach repeats in its extracellular domain, DOES NOT MAKE INFECTIONS 2 (DMI2), and the putative cation channel DOES NOT MAKE INFECTIONS 1 (DMI1), which are important for the activation of calcium spiking in the perinuclear zone and nucleus. In contrast, calcium influx and subsequent deformations of root hairs are not dependent on DMI2 and DMI1 (Esseling et al., 2004). Similarly, in the *symrk* (orthologous to DMI2), *castor, pollux* (orthologous to DMI1) (Ané et al., 2004; Imaizumi-Anraku et al., 2005) *Lotus japonicus* mutants with disturbed calcium spiking, nevertheless, the calcium influx and deformation of root hairs were observed (Miwa et al., 2006). Finally, both pathways result in the phosphorylation of calcium and calmodulin-dependent kinase, DOES NOT MAKE INFECTIONS 3 (DMI3), which stimulates a complex of transcription factors required for infection thread development and nodule organogenesis.

Activation of differing signaling pathways may be related to the involvement of two receptor complexes in legume plants that regulate calcium influx and calcium spiking (Geurts et al., 2005). It is interesting to note that the responses induced by the G-protein complex agonist mastoparan also occurred in *dmi2* and *dmi1 M. truncatula* mutants, but these responses were not observed in the *dmi3* mutant (den Hartog et al., 2003). The lack of dependence on DMI2 and DMI1 suggests that the heterotrimeric G-protein complex may be involved in the activation of calcium influx, followed by root hair deformation. This hypothesis will need to be tested in future studies and requires more precise analysis of G-protein subunits interaction with kinase domains of receptors to Nod factors. In our experiments the interaction between PsG alpha and kinase domain of LysM-RLK K1 was shown. Since LysM-RLK K1 controls the earliest reactions like deformations after signal perception in pea plants, further analysis of *k1* mutants should be performed to obtain evidences of G-protein involvement in signal transduction.

## Data Availability Statement

The datasets presented in this study can be found in online repositories. The names of the repository/repositories and accession number(s) can be found in the article/Supplementary Material.

## Author Contributions

AB: investigation, writing original draft preparation, and methodology. OP: investigation, heterologous protein synthesis, and co-immunoprecipitation. AD: genome-based searching and analysis and graphic output of phylogenetic tree. IL: methodology, cloning, and plant transformation. ED: conceptualization, writing and editing, and supervision. All authors have read and agreed to the manuscript.

## Conflict of Interest

The authors declare that the research was conducted in the absence of any commercial or financial relationships that could be construed as a potential conflict of interest.

## Publisher’s Note

All claims expressed in this article are solely those of the authors and do not necessarily represent those of their affiliated organizations, or those of the publisher, the editors and the reviewers. Any product that may be evaluated in this article, or claim that may be made by its manufacturer, is not guaranteed or endorsed by the publisher.

## Supplementary Material

Supplementary Figure 1 Phylogenetic tree constructed using the Maximum-Likelihood method based on amino acid sequences of *G. max G alpha* and *A. thaliana G alpha* and *XLG* genes and their homologous identified in *P. sativum* and *M. truncatula* genomes. Previously identified and exposed in NCBI *PsGalpha1* (AF537218) and *PsGalpha2* (AF533438) were also included in this tree. Numeric values indicate branch support based on 1000 UltraFast bootstrap replicates.

Supplementary Figure 2 Phylogenetic tree constructed using the Maximum-Likelihood method based on amino acid sequences of *G. max* and *A. thaliana Ggamma* genes and their homologous identified in *P. sativum* and *M. truncatula* genome. Numeric values indicate branch support based on 1000 UltraFast bootstrap replicates.

Supplementary Figure 3 **(A)** Heatmap shows log-tranformed CPM values of *Gbeta*, *Galpha*, *XLG* and *Ggamma* genes expression in *M. truncatula* during early stages of symbiosis development based on Gene expression data from GSE133612 project (Schiessl et al., 2019). **(B)** Bar plots represent CPM values of *Gbeta, Galpha, XLG* and *Ggamma* gene expression in *M. truncatula* nodules and non-inoculated roots (Roux et al., 2014).

Supplementary Figure 4 Composite plants of *M. truncatula* A17 **(A,B)** and *P. sativum* cv. Finale **(C,D)** plants with the *Gbeta1* gene suppression in transgenic roots (*Gbeta*-RNAi) **(B,D)** and control plants with β-glucuronidase gene overexpression (*GUS*-OE) **(A,C)**. The number of nodules were scored only in transgenic fluorescent roots.

Supplementary Figure 5 Bar plots represent CPM values of *MtPLC* genes expression in *M. truncatula* during early stages of symbiosis development based on Gene expression data from GSE133612 project (Schiessl et al., 2019).

Supplementary Figure 6 Bar plots represent CPM values of *MtPLD* genes expression in *M. truncatula* during early stages of symbiosis development based on Gene expression data from GSE133612 project (Schiessl et al., 2019).

Supplementary Table 1 List of primers used in this study.

Supplementary Table 2 The genome of M. truncatula encodes nine PLC and fifteen PLD genes. Analysis of transcriptomic datasets for M. truncatula roots inoculated with rhizobia, or treated with Nod factors factors (van Zeijl et al., 2015; Damiani et al., 2016; Schiessl et al., 2019), revealed that the expression of MtPLC1 (Medtr3g070560) (Damiani et al., 2016; Schiessl et al., 2019) and MtPLD1 (Medtr4g010650) increased at the early stages of symbiosis development (Supplementary Figures 4, 5). Genes in P. sativum were selected based on homology to the orthologs of M. truncatula and included one PsPLC1 (Psat5g128400) gene and one PsPLD1 (Psat7g255400) gene.
