# Supplementary figures and images for: The Role of Heterotrimeric G-Protein Beta Subunits During Nodulation in Medicago truncatula Gaertn and Pisum sativum L
Source: Front Plant Sci. 2022 Jan 12;12:808573. doi: 10.3389/fpls.2021.808573 (PMC8790031; doi:10.3389/fpls.2021.808573)

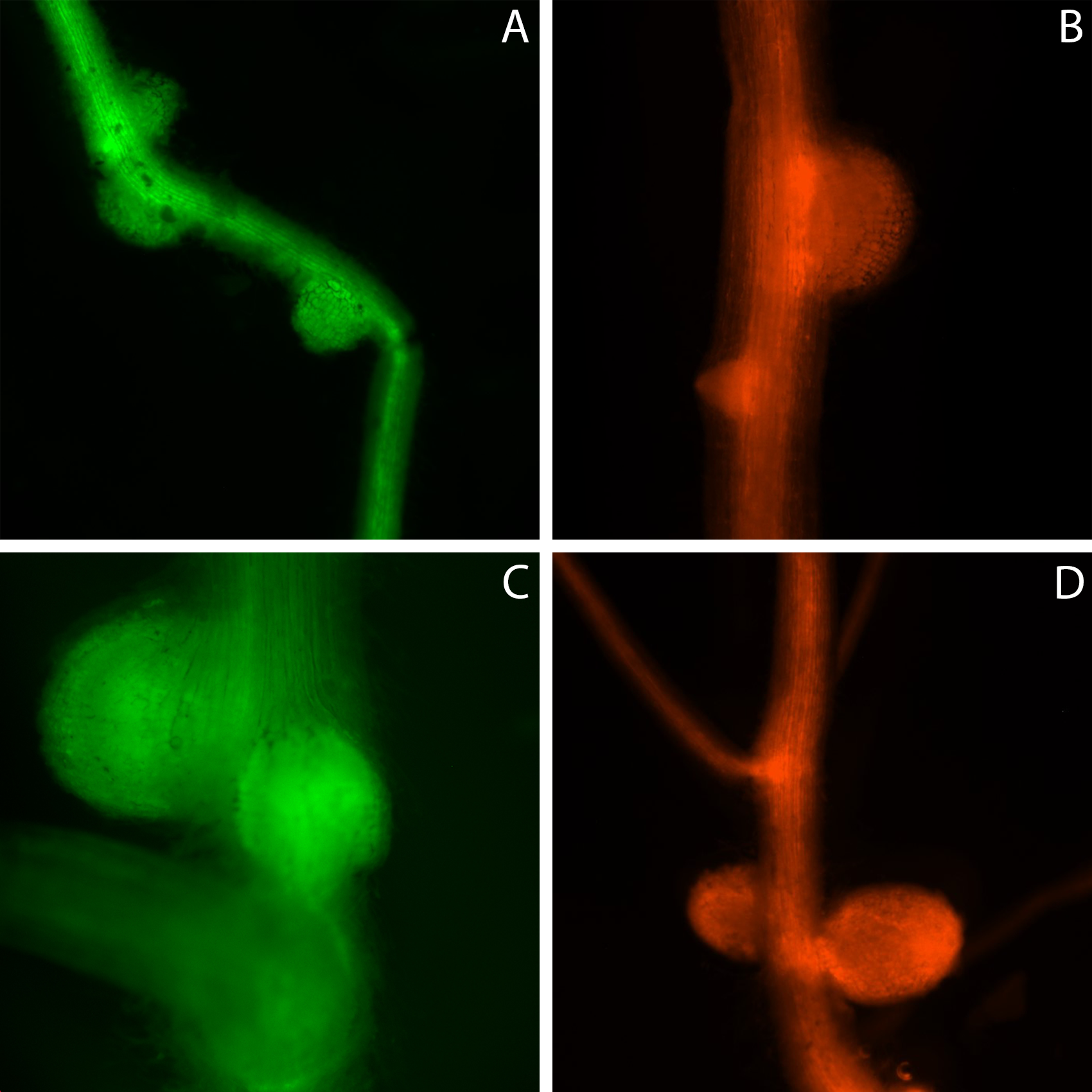

Supplement: Supplementary Figure 1 — Phylogenetic tree constructed using the Maximum-Likelihood method based on amino acid sequences of G. max G alpha and A. thaliana G alpha and XLG genes and their homologous identified in P. sativum and M. truncatula genomes. Previously identified and exposed in NCBI PsGalpha1 (AF537218) and PsGalpha2 (AF533438) were also included in this tree. Numeric values indicate branch support based on 1000 UltraFast bootstrap replicates. [file Image_1.TIF]

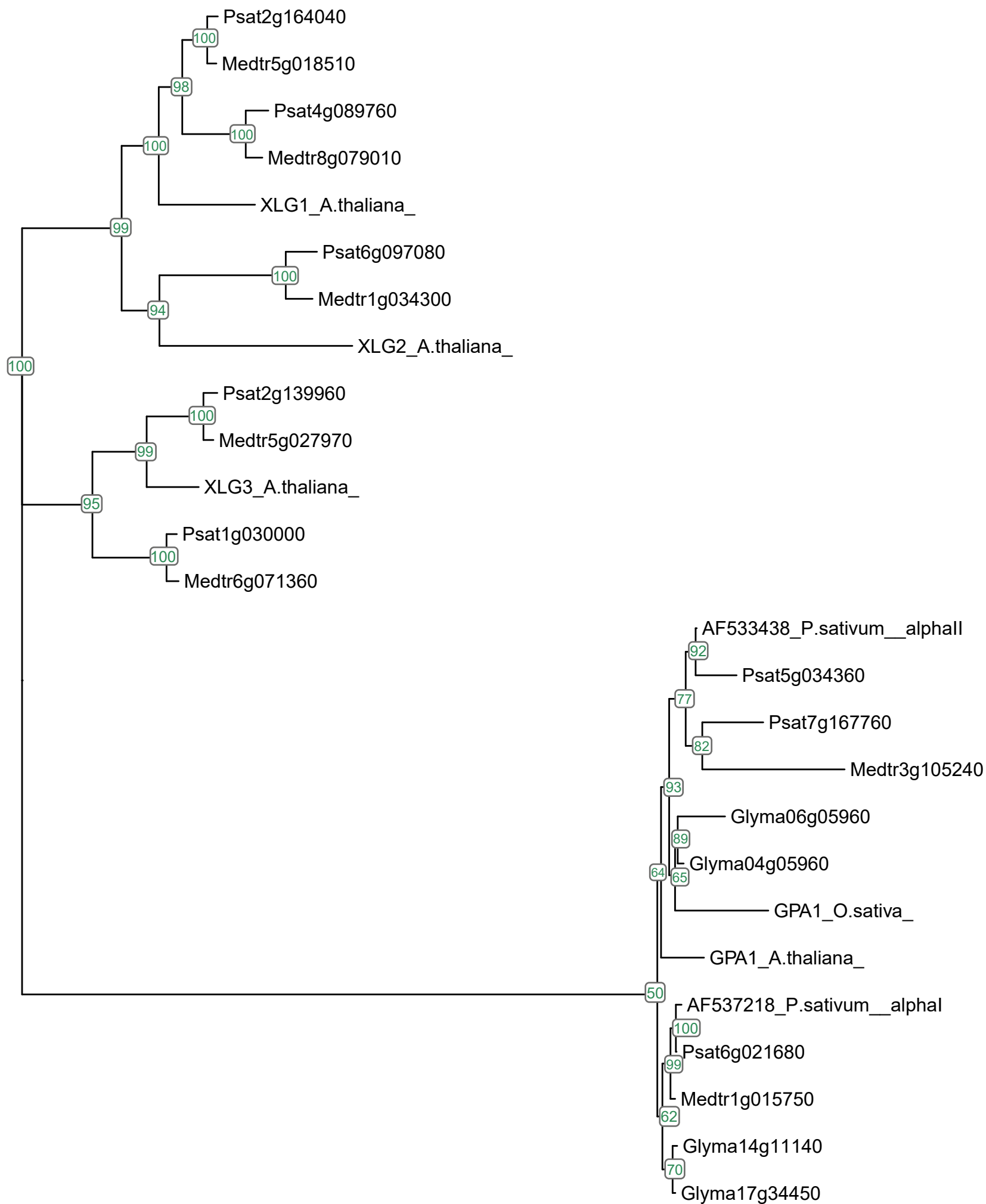

Supplement: Supplementary Figure 2 — Phylogenetic tree constructed using the Maximum-Likelihood method based on amino acid sequences of G. max and A. thaliana Ggamma genes and their homologous identified in P. sativum and M. truncatula genome. Numeric values indicate branch support based on 1000 UltraFast bootstrap replicates. [file Data_Sheet_1.PDF]

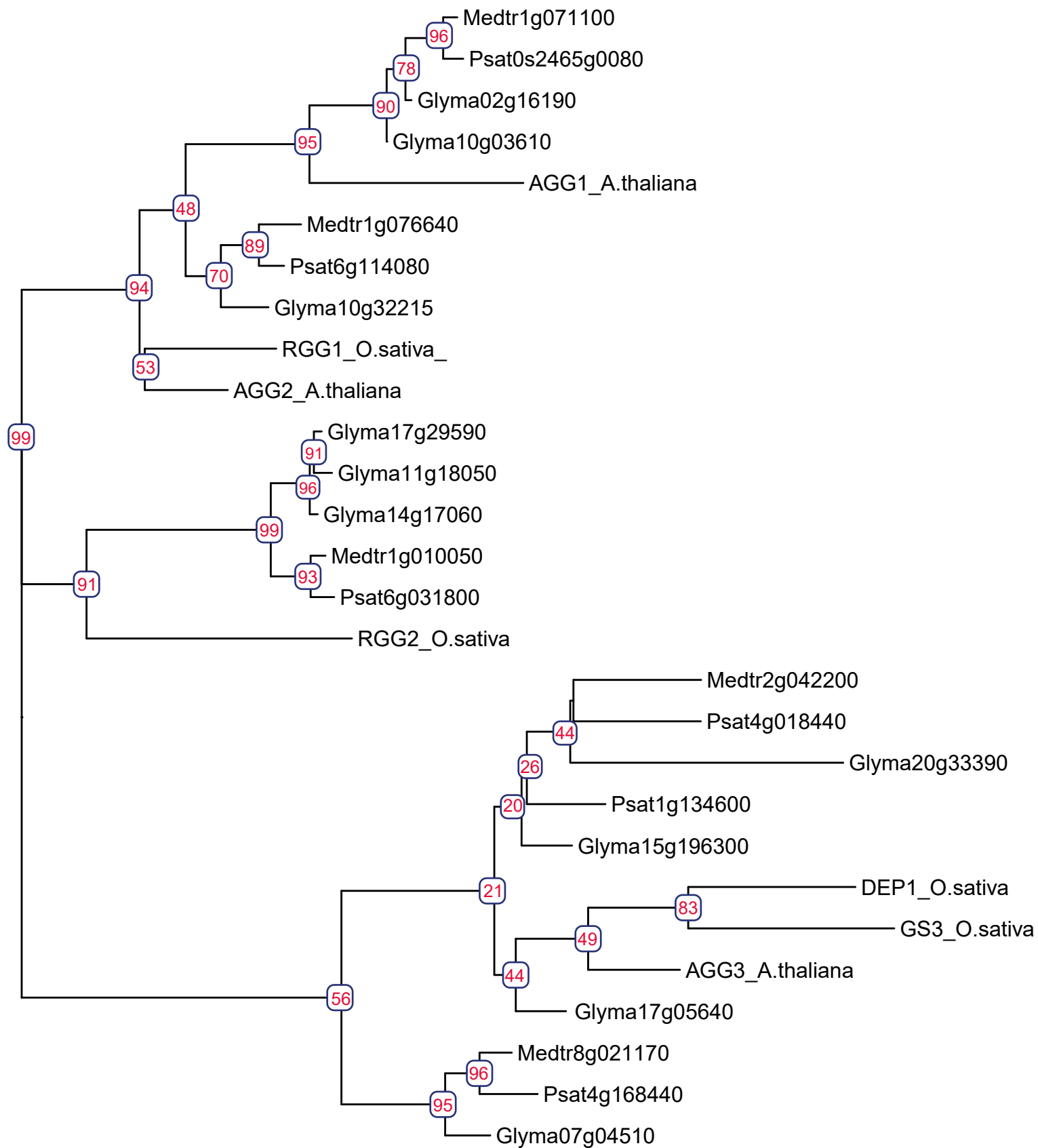

Supplement: Supplementary Figure 3 — (A) Heatmap shows log-tranformed CPM values of Gbeta, Galpha, XLG and Ggamma genes expression in M. truncatula during early stages of symbiosis development based on Gene expression data from GSE133612 project (Schiessl et al., 2019). (B) Bar plots represent CPM values of Gbeta, Galpha, XLG and Ggamma gene expression in M. truncatula nodules and non-inoculated roots (Roux et al., 2014). [file Data_Sheet_2.PDF]

A

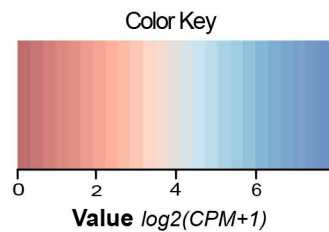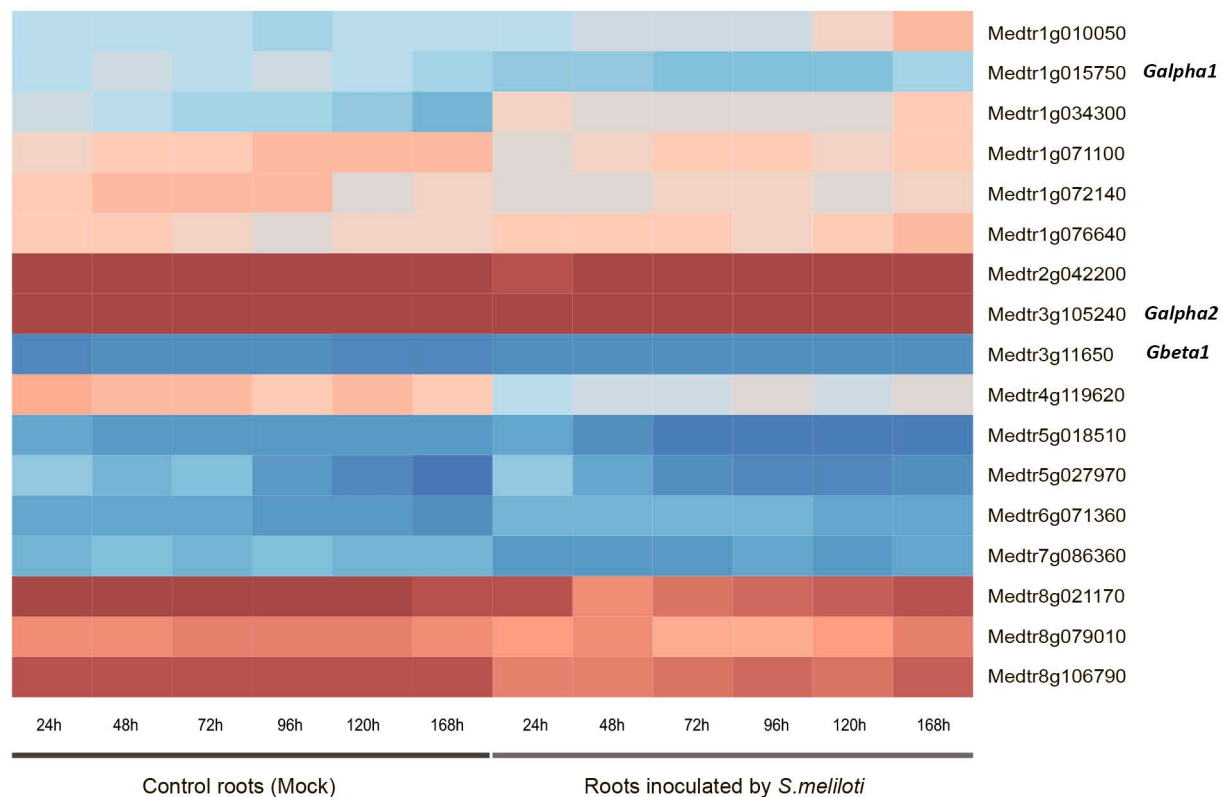

B

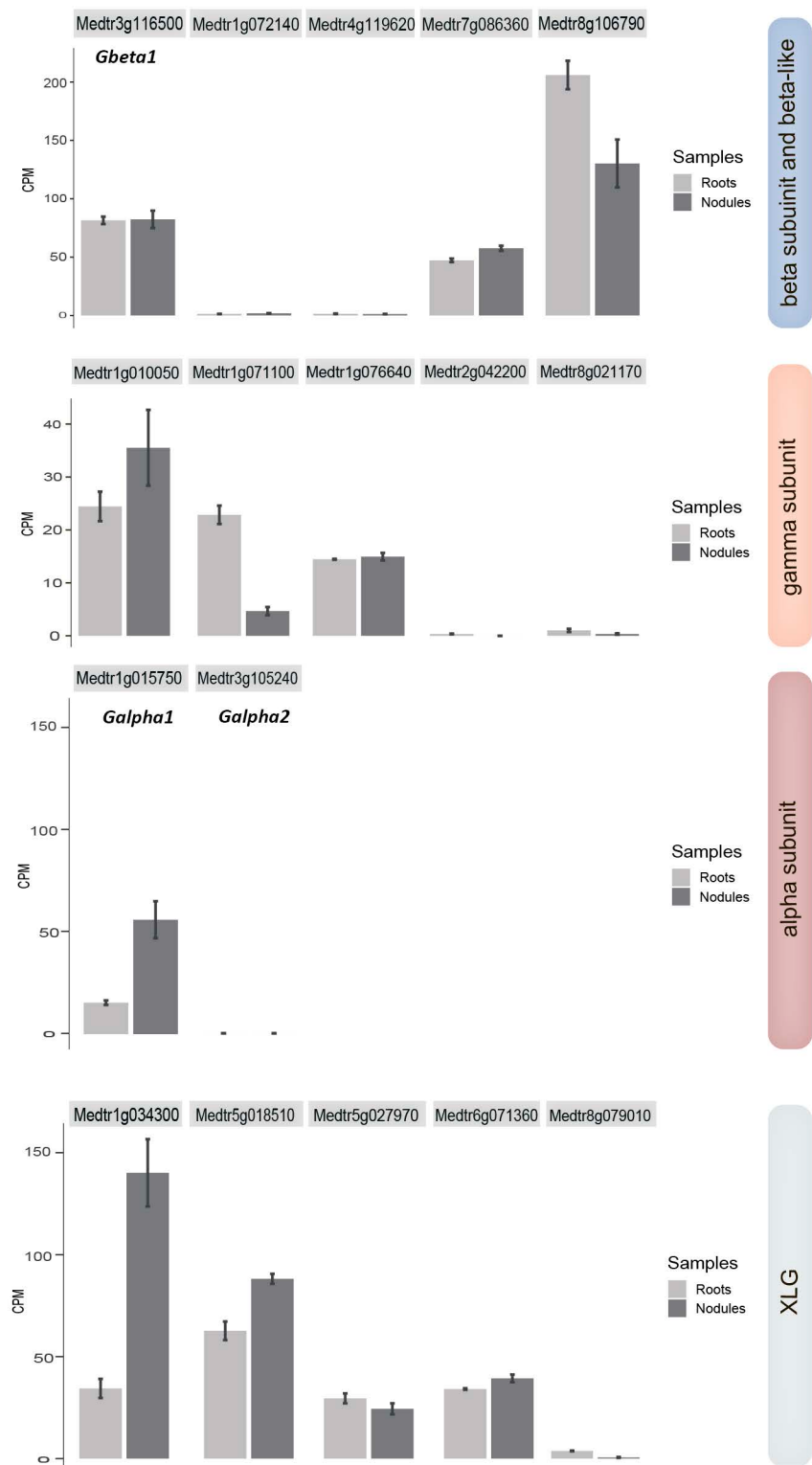

Supplement: Supplementary Figure 4 — Composite plants of M. truncatula A17 (A,B) and P. sativum cv. Finale (C,D) plants with the Gbeta1 gene suppression in transgenic roots (Gbeta-RNAi) (B,D) and control plants with β-glucuronidase gene overexpression (GUS-OE) (A,C). The number of nodules were scored only in transgenic fluorescent roots. [file Data_Sheet_3.PDF]

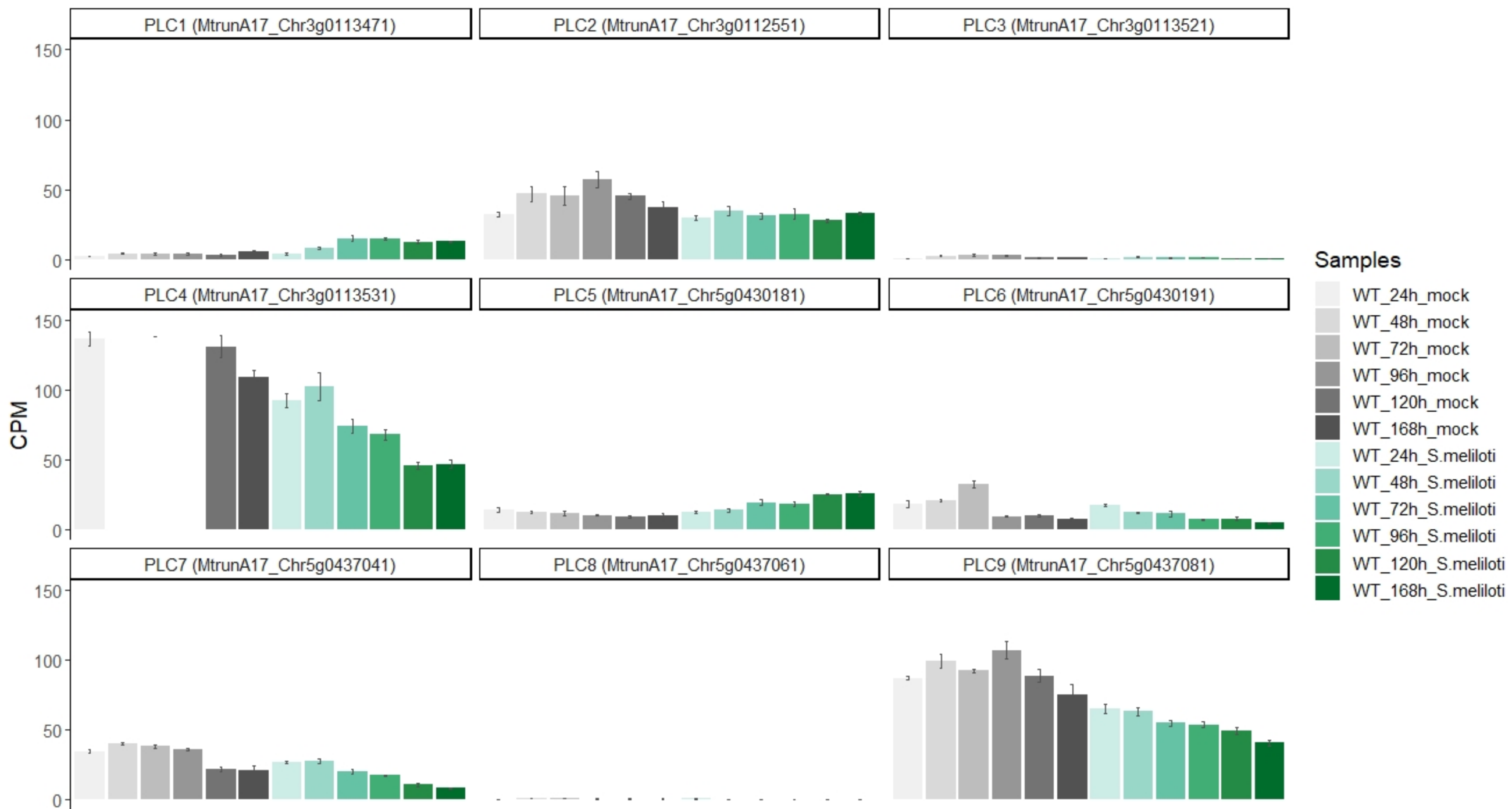

Supplement: Supplementary Figure 5 — Bar plots represent CPM values of MtPLC genes expression in M. truncatula during early stages of symbiosis development based on Gene expression data from GSE133612 project (Schiessl et al., 2019). [file Data_Sheet_4.PDF]

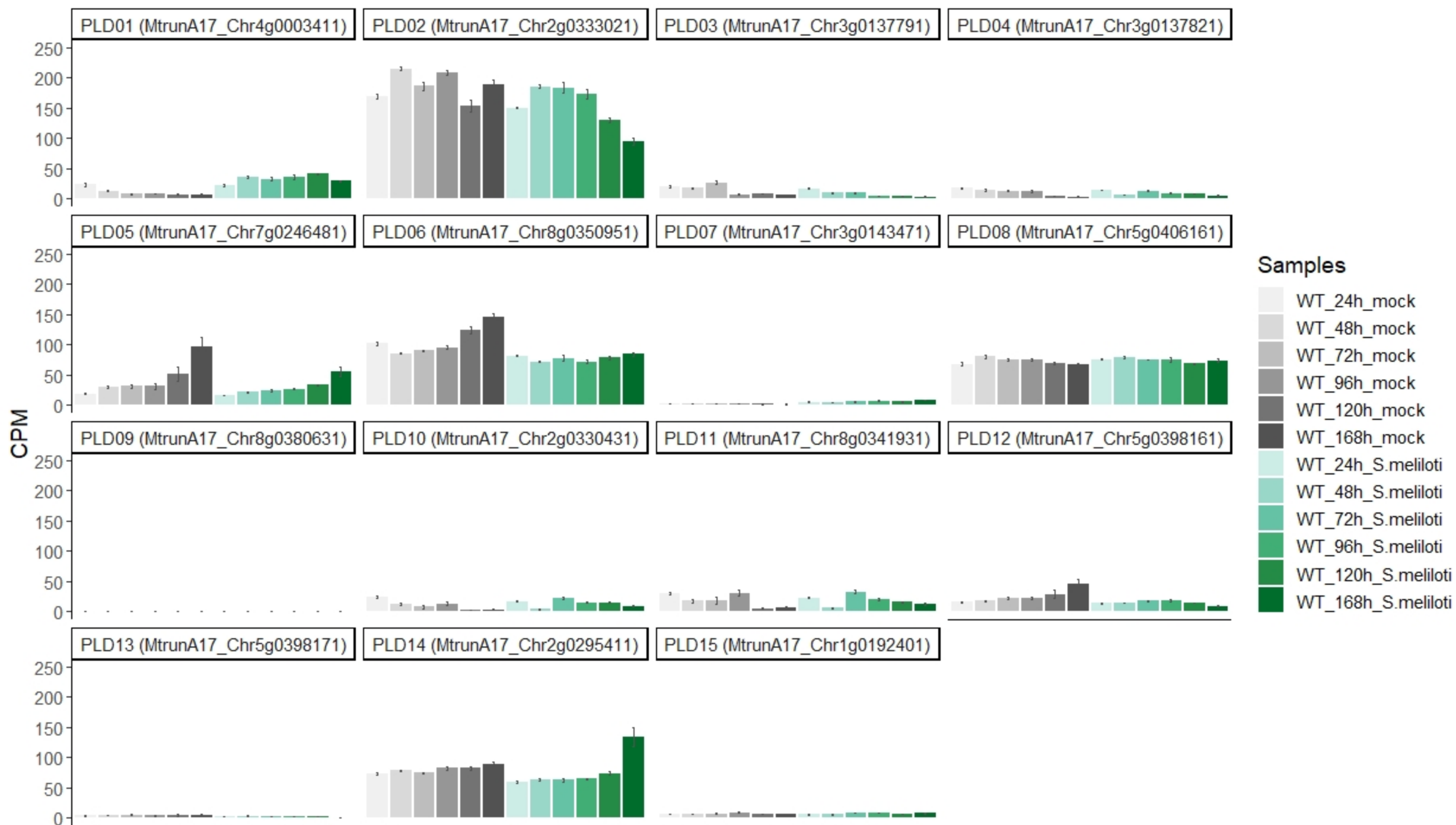

Supplement: Supplementary Figure 6 — Bar plots represent CPM values of MtPLD genes expression in M. truncatula during early stages of symbiosis development based on Gene expression data from GSE133612 project (Schiessl et al., 2019). [file Data_Sheet_5.PDF]
